# Differential Matrix Metalloprotease (MMP) Expression Profiles Found in Aged Gingiva

**DOI:** 10.1371/journal.pone.0158777

**Published:** 2016-07-08

**Authors:** Suhee Kim, Sun Hee Ahn, Jin-Sil Lee, Ji-Eun Song, Sung-Hyun Cho, Seunggon Jung, Seon-Kyu Kim, Seok-Ho Kim, Kwang-Pyo Lee, Ki-Sun Kwon, Tae-Hoon Lee

**Affiliations:** 1 Department of Oral Biochemistry, Dental Science Research Institute, Medical Research Center for Biomineralization Disorders, School of Dentistry, Chonnam National University, Gwangju, Republic of Korea; 2 Department of Molecular Medicine (BK21plus), Chonnam National University Graduate School, Gwangju, Republic of Korea; 3 Department of Oral and Maxillofacial Surgery, School of Dentistry, Chonnam National University, Gwangju, Republic of Korea; 4 Personalized Genomic Medicine Research Center, Korea Research Institute of Bioscience and Biotechnology (KRIBB), Daejeon, Republic of Korea; 5 Aging Research Center, Korea Research Institute of Bioscience and Biotechnology (KRIBB), Daejeon, Republic of Korea; INRS, CANADA

## Abstract

The periodontium undergoes age-related cellular and clinical changes, but the involved genes are not yet known. Here, we investigated age-related genetic changes in gingiva at the transcriptomic level. Genes that were differentially expressed between young and old human gingiva were identified by RNA sequencing and verified by real-time PCR. A total of 1939 mRNA transcripts showed significantly differential expression between young and old gingival tissues. Matrix metalloprotease (MMP) regulation was the top pathway involved in gingival aging. MMP3, MMP9, MMP12, and MMP13 were upregulated in old gingival tissues, concomitantly with interleukin-1 beta (IL1B) expression. In vitro experiments using human gingival fibroblasts (hGFs) showed that MMP12 was upregulated in old hGFs compared to young hGFs. Moreover, the MMP3, MMP9 and IL1B levels were more highly stimulated by infection with the oral bacterium, *Fusobacterium nucleatum*, in old hGFs compared to young hGFs. Collectively, these findings suggest that, in gingiva, the upregulation of MMP12 may be a molecular hallmark of natural aging, while the upregulations of MMP3, MMM9, and IL1B may indicate externally (e.g., infection)-induced aging. These findings contribute to our understanding of the molecular targets involved in gingival aging.

## Introduction

Aging gradually impairs most tissues, including the periodontium [1, 2]. The periodontium consists of the gingiva, the periodontal ligament, the cementum, and the alveolar bone, which supports the teeth [3]. The gingiva is the part of the oral mucosa that covers the alveolar processes of the jawbones and surrounds the necks of the teeth [4]. Histologically, it is formed by epithelia and connective tissues [3]. As age increases, the gingiva shows clinical reductions in its structures and resilience, migrates toward the apex, and becomes more sensitive to external factors present in the oral cavity [2].

Cellular changes have been reported in aged gingiva, including: diminished keratinization in the epithelium; modification of the junctions between the epithelium and connective tissues; decreased cell numbers in connective tissues; and morphological/functional alterations of fibroblasts in the gingiva [5–9]. A considerable amount of data is available on biological effect of aging in the periodontium [2, 10], but very few studies have investigated age-related genetic changes in the gingiva [11], and specific genes have not been comprehensively understood for gingival aging.

Older persons have an increased risk for chronic diseases of the mouth, including dental infections and oral cancer [12]. Recently, increasing attention has focused on identifying the cellular and molecular changes of the periodontium that form the basis for the high prevalence of oral diseases in aged populations [13]. The study of molecular changes in aged gingiva under pathological oral conditions may enable us to recognize and assess differences between natural aging and inflamm-aging, and improve our ability to help older patients maintain good oral health.

The present study sought to: i) use RNA sequencing to identify genes that showed significant differential expression between young and old gingiva; ii) identify genes that are involved in gingival aging; and iii) determine whether the identified genes are associated with internal (natural) or external (stimulant-induced) aging by comparing their expression levels in human old and young gingival fibroblasts (hGFs) with or without infection by oral bacteria. The obtained results would contribute to our understanding of the potential for normal aging biomarkers and markers driven by pathogenic conditions (*e*.*g*., xerostomia, inflammation and infections) to be used as aging-related risk factors in gingiva.

## Materials and Methods

### Ethics Statement

Collection of human gingival tissue was approved by the IRB in Chonnam National University Dental Hospital (approved No.CNUDH-2013-001). Gingival tissue biopsies from human patients were collected with informed consent from Chonnam National University Dental Hospital of Korea between 2013 and 2015. Written informed consent was obtained for all subjects after the nature and possible consequences of the studies were explained. All participants were adults without periodontal disease.

### Gingival tissue samples

The inflammatory status of each gingival tissue sample was assessed by a dentist, and biopsies from sites judged as healthy were included in this study. Biopsies were stored in liquid nitrogen (LN_2_) immediately after collection. Information on the samples is provided in S1 Table.

### Culture of primary human oral cells

Primary human gingival fibroblasts (hGFs) were prepared from gingival tissues of adult participants without periodontal disease, as described by Min et al. [14]. hGFs were grown in Dulbecco's modified Eagle’s medium (DMEM; Gibco BRL, Grand Island, NY, USA) supplemented with 10% heat-inactivated fetal bovine serum (PAA Laboratories, Etobicoke, Ontario, Canada), 100 U/ml penicillin and 100 μg/ml streptomycin (Gibco BRL) at 37°C in a humidified atmosphere containing 5% CO_2_. When confluent, the cells were trypsinized using 0.25% trypsin/0.02% EDTA solution (Sigma, St. Louis, MO, USA). *In vitro* aging of hGFs was performed by sequential subcultivations (young, 4 population doublings; old, 22 population doublings) and validated using β-galactosidase staining to detect senescent cells.

### Bacterial strain and culture

*Fusobacterium nucleatum* subsp. polymorphum ATCC10953 was cultured in an anaerobic chamber (85% N_2_, 5% CO_2_ and 10% H_2_) at 37°C in tryptic soy broth (TSB) supplemented with 5 μg/ml hemin (Sigma) and 1 μg/ml menadione (Sigma). Bacteria were collected by centrifugation (7,000 × g, 10 min), washed in phosphate-buffered saline (PBS), and adjusted to 10^8^ CFU/ml in buffer based on matching the optical density (OD) to a reference standard. For enumeration, bacteria were subjected to anaerobic culture in TSB supplemented with 1.5% agar (Sigma), 5% sterile defibrinated sheep blood, 5 μg/ml hemin and 1 μg/ml menadione.

### Bacterial infection

Young and old hGFs were infected with *F*. *nucleatum* (MOI = 10) and incubated for 0.5, 2, 6, and 12 h. The cells were harvested at the indicated time points for RNA extraction.

### RNA extraction

Total RNA was isolated using an RNeasy kit (Qiagen, Valencia, CA, USA) according to the manufacturer's instructions. RNA quantity and quality were assessed with an Epoch Microplate Spectrophotometer (Biotek^®^, Winooski, VT, USA).

### RNA sequencing and functional annotation

For each total RNA sample, 4 μg was subjected to purification of poly-A transcripts and generation of libraries with multiplexed barcode adaptors, using the appropriate TrueSeq sample preparation kits (Illumina, San Diego, CA, USA). All samples passed quality control analysis on a Bioanalyzer 2100 (Agilent Technologies, Santa Clara, CA, USA), and paired-end reads (2 × 100 bp) were generated with a HiSeq 2000 sequencing system (Illumina).

Reference genome sequence data from Homo sapiens were obtained from the University of California Santa Cruz Genome Browser Gateway (assembly ID: hg19). The reference genome index was built using the Bowtie2-build component of Bowtie2 (ver. 2.0) and SAMtools (ver. 0.1.18). Tophat2 (ver. 2.0) was applied to map the reads obtained from our tissue samples to the reference genome. The fragments per kilobase of transcript per million mapped reads (FPKM) values were used to estimate the expression level of each gene. The FPKM data were normalized by the quantile method, log2-transformed, and median-centered across genes and samples. Using normalized FPKM data, we applied a hierarchical cluster analysis, taking the centered correlation coefficient as the measure of similarity and performing complete linkage clustering. To assess the significance of differences in gene expression between sample groups, we used the EdgeR package with a negative binomial model to select differentially expressed genes from the count data. Differences in gene expression were considered statistically significant if the *p* value was < 0.001 and the fold difference in expression between two sample groups was ≥ 2. Gene set enrichment analysis was performed using the Ingenuity Pathway tool (ver. 8.0; Ingenuity Systems, Redmond City, CA, USA), and the significance of over-represented gene sets was estimated using Fisher’s exact test.

### Real-time PCR

The amplified genes and utilized primers are listed in S2 Table. Total RNA was reverse-transcribed using a PrimeScript^™^ RT Reagent Kit (TaKaRa, Tokyo, Japan) according to the manufacturer's instructions. Real-time quantitative PCR was performed using an ABI Prism 7300 Sequence Detection System (Applied Biosystems, Foster City, CA, USA) and the SYBR Green Master Mix (TaKaRa). All data were normalized with respect to β-actin, and expressed as the relative fold ratio for control (young sample) by a formulation using 2^-ΔΔ^CT method.

### Zymography

The activity of proteolytic enzymes was evaluated using gelatin or casein zymography following the electrophoretic separation of lysates from young and old human gingival tissues (n = 3 individuals per group; S1 Table). Briefly, gingival tissues (30 mg) were mixed with homogenization buffer (50 mM Tris-HCl, pH 6.8, 150 mM NaCl, and 1% Triton X-100) and homogenized with a pestle on ice, and lysates were collected by centrifugation (5,000 × g, 10 min, 4°C). Twenty micrograms of unheated and non-denatured protein were subjected to 0.1% gelatin (Sigma) or 0.1% β-casein (Sigma)-containing SDS-polyacrylamide gel electrophoresis. Gels were washed in renaturing buffer (2.5% Triton X-100) and incubated in Novex zymogram developing buffer (Invitrogen, Carlsbad, CA, USA) for 16 h at 37°C. Gels were stained with 0.2% Coomassie brilliant blue R (Sigma) for 1 h and then destained in 20% methanol and 10% glacial acetic acid. Gelatinolytic and caseinolytic activities were detected as white areas against a blue background. The areas were quantified with the CS Analyzer 4.0 software (ATTO Co., Tokyo, Japan).

### Statistics

Statistical analysis was performed using the SPSS 17.0 software package (SPSS, Chicago, IL, USA). All data were analyzed using t tests, values of *p* < 0.05 were considered significant.

## Results

### RNA sequencing

We sequenced the transcriptomes of human gingival tissues obtained from three young individuals (17–20 years old) and three old individuals (≥ 60 years old). Alignment of the obtained sequence reads against the human genome yielded a concordant pair alignment rate > 80% across all samples (S3 Table). Biological replicates had high correlation coefficients for gene expression both within each group and all samples (*r* > 0.89), indicating that the gene expression patterns showed little variation among samples (S1 Fig).

### Distribution of gene transcripts between young and old gingival tissues

**Fig 1:**
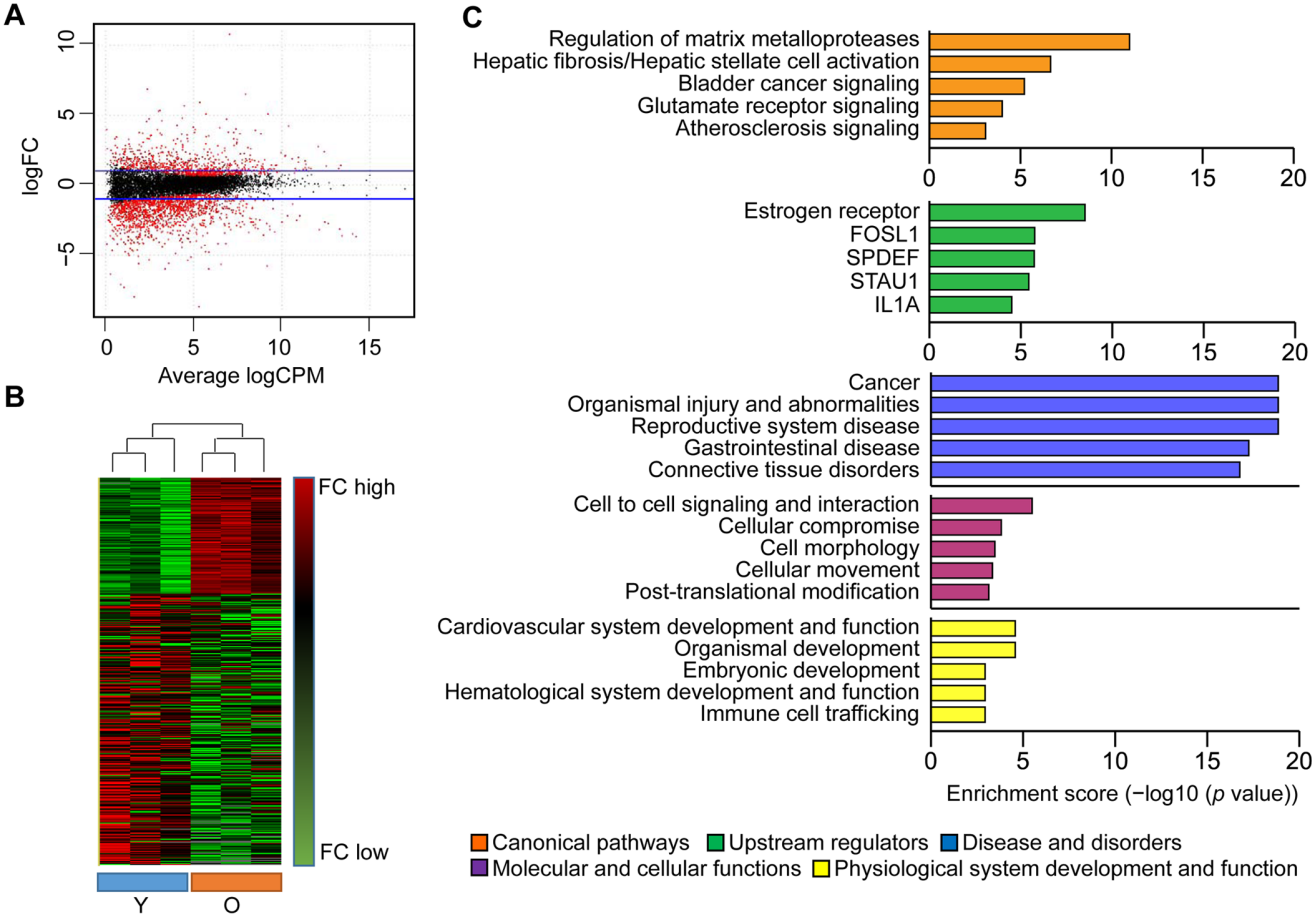
Analysis of gene transcripts in young and old gingival tissues using RNA sequencing. (A) Genes with significantly differential expression between young and old gingival tissues were shown as scatter plot. Blue lines represent baseline of upregulated and downregulated genes in old gingival tissues versus young gingival tissues in the range of ≥ ±2 fold differences, respectively. (B) Hierarchically heat map of differential expressed genes was presented for each individuals. Highly expressed genes in young or old were displayed as red, while lower expressed genes as green. (C) Ingenuity Pathway Analysis (IPA) were performed among the DEGs for pathways, upstream regulators, diseases and bio functions. Top 5 ranking per each categories is shown as enrichment score (−log10 (*p* value)). Y, young gingival tissues; and O, old gingival tissues.

Differentially expressed genes (DEGs) are presented as scatter plots (Fig 1A) and hierarchically clustered heatmap for all tissue samples (Fig 1B). Hierarchically clustered heatmap revealed that the dendrogram of samples was divided into two parts (young and old groups). The majority of the young group clustered together and the majority of the old group also clustered together based on the correlation between samples’ gene expression. In total, 1939 mRNA transcripts showed significant differential expression between the young and old gingival tissue samples. Of them, 591 were upregulated and 1348 were downregulated in old versus young gingival tissue samples (S4 Table).

DEGs showing changes of 4-fold or more were subjected to Ingenuity Pathway Analysis, in an effort to identify canonical pathways, upstream regulators, diseases and bio-functions that may be closely related to gingival aging (Fig 1C). The top canonical pathway found to be differentially expressed between old and young gingival tissues was the regulation of matrix metalloproteases (MMPs); the top activated regulators included estrogen receptor, SAM pointed domain-containing Ets transcription factor (SPDEF), and interleukin-1 alpha (IL1A); and the inhibited regulator was double-stranded RNA binding protein Staufen homolog 1 (STAU1). The full DEG list and the results of our gene set enrichment analysis are provided in S5 Table.

### MMPs activated in old gingival tissues

**Fig 2:**
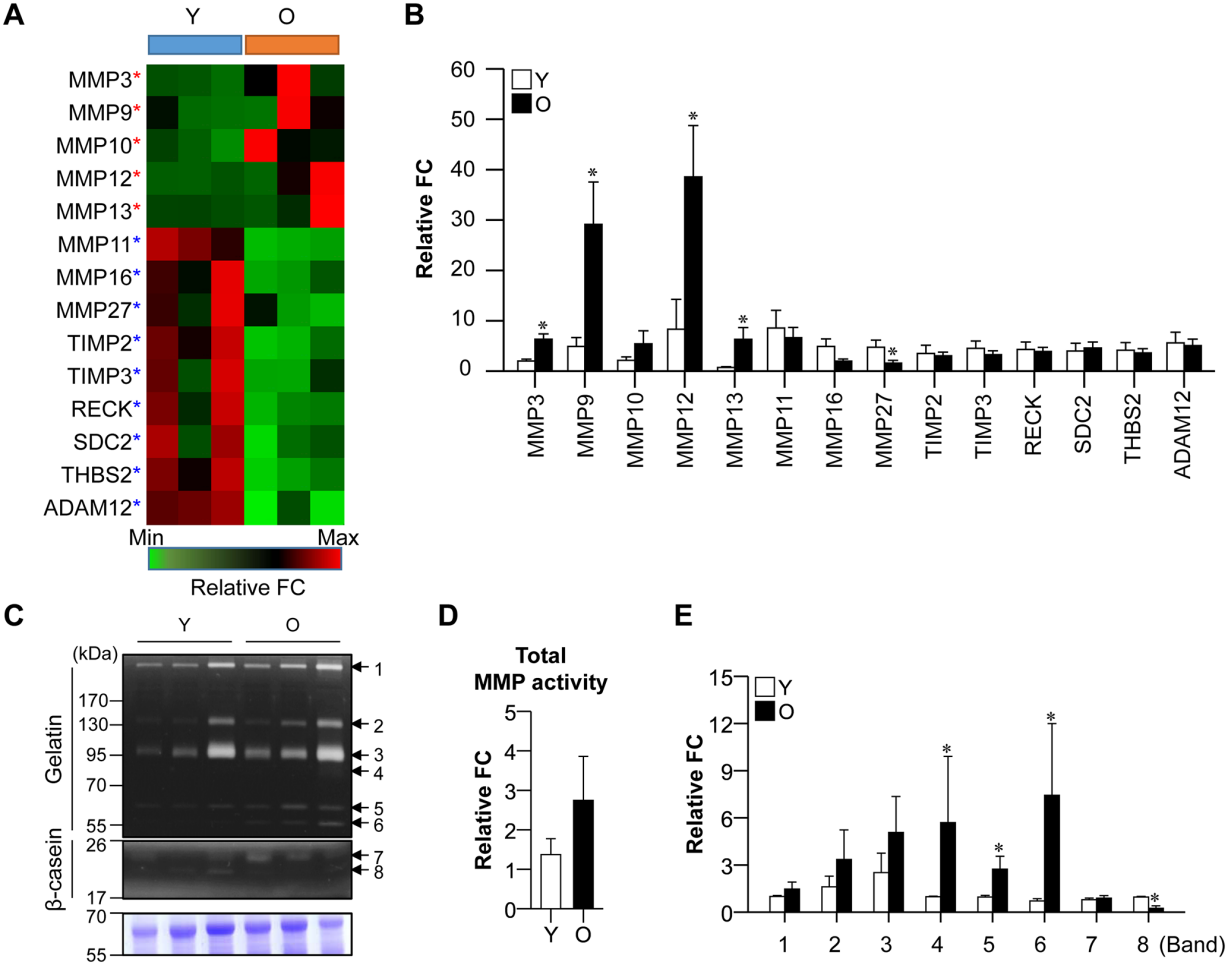
Expression of MMP regulation pathway-related genes in aged gingival tissues. (A) RNA sequencing of MMP regulation-related genes in young and old gingival tissues (n = 3 per group). Red and green stars indicate mRNA that were up- and downregulated, respectively, in old gingival tissues. Red color indicates high-level gene expression in young or old gingival tissues. (B) Confirmation of RNA sequencing results using real-time PCR (n = 5 per group; *p < 0.05). (C) MMP activity was assessed by zymography. (D and G) Total MMP activity (D) and specific MMP activities (G) were calculated according to the density of each protein band. Y, young gingival tissues; and O, old gingival tissues.

We investigated the available reports on gene categories involved in aged tissues. From among the top five canonical pathways believed to be affected by age, we focused on the MMPs, which are critical for gingival wound healing and were recently considered to be severely affected by aging [15]. To test whether genes associated with MMP regulation were altered under gingival aging, we used real-time PCR to validate the mRNA levels of selected genes in young and old gingival tissues (n = 5 per group), in comparison with our RNA sequencing data. Of 14 genes related to MMP regulation, MMP3, MMP9, MMP12, and MMP13 were significantly upregulated and MMP27 was downregulated in old versus young gingival tissues, as assessed by both RNA sequencing (Fig 2A) and real-time PCR (Fig 2B). However, there was no significant different in the expression levels of the tissue inhibitors of MMPs (TIMP2 and TIMP3) between old and young gingival tissues in our real-time PCR data.

To determine whether the differential mRNA expression of MMPs in aged gingival tissues was associated with changes in their protein activity, we assessed the proteolytic activity of MMPs. Eight MMP protein bands showing gelatinolytic and caseinolytic activity were detected in gingival tissues (Fig 2C). Although there was little difference in total MMP activity between young and old gingival tissues (Fig 2D), consideration of the molecular sizes and substrates suggested that the activities of MMP9 (band 4, 86 kDa), ProMMP13 (band 5, 60 kDa), and ProMMP3 (band 6, 57 kDa) were increased, while that of MMP7 (band 8, 19 kDa) was decreased in old gingival tissues (Fig 2E). There was no significant difference in the activity of MMP complexes (band 1 and 2, 130 and 250 kDa), ProMMP9 (band 3, 92 kDa), and MMP12 (band 7, 22 kDa). These data suggest that gingival tissues can produce excessive activity of specific MMPs. This might decrease the ability of tissues to maintain tissue homeostasis, which may be indicative of gingival tissue aging.

### MMP-associated network genes in old gingival tissues

**Fig 3:**
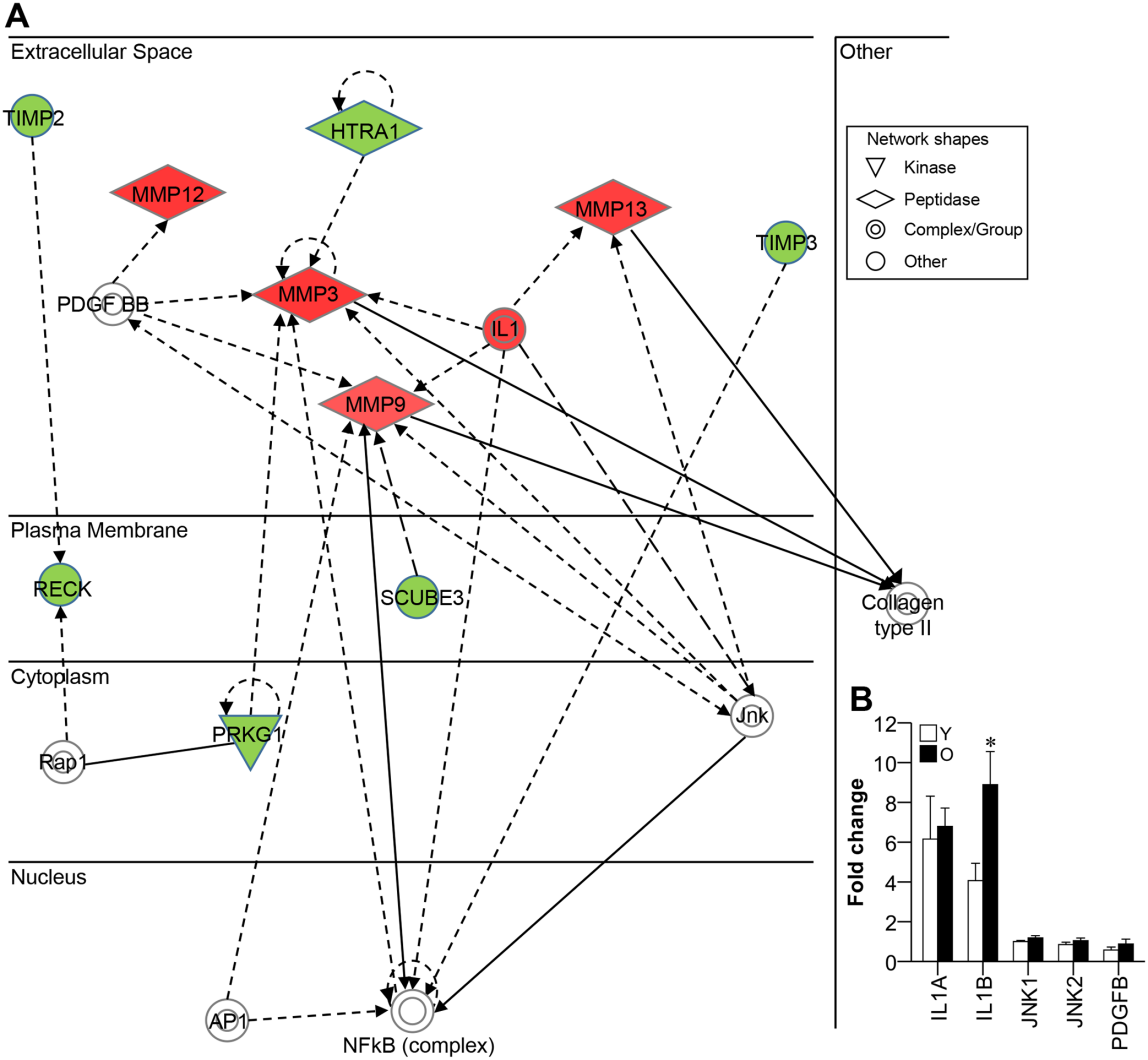
Network of genes co-expressed with MMPs. (A) Genes that were co-regulated with the MMPs that were upregulated in old gingival tissues (red diamond shapes) were analyzed using IPA. Highly expressed genes in old gingival tissue were filled with red, while lower expressed genes as green. Genes filled with white indicate no significant difference between young and old gingival tissues, but are incorporated into the network through relationships with other molecules. (B) The several genes (IL1, JNK, and PDGF BB complexes) that were associated with MMP regulation were identified in old gingival tissues using real-time PCR. Y, young gingival tissues; and O, old gingival tissues; n = 5; and *p < 0.05 versus the results from Y.

Next, we checked what is associated with triggering of MMPs (MMP3, -9, -12, and -13) by analyzing network of molecules known to co-express with MMPs or regulate MMPs using Ingenuity Pathway Analysis (IPA) (Fig 3A). As shown in Fig 3A, several genes such as IL1, platelet-derived growth factor-BB (PDGFBB) and c-Jun N-terminal kinase (JNK), appeared to be involved in the upregulation of MMPs. We thus used real-time PCR to examine the mRNA expression levels of IL1A, IL1B, PDGFB, JNK1 and JNK2 in young and old gingival tissues. Our results revealed that IL1B was upregulated in old gingival tissues compared to young gingival tissues, whereas the other candidate genes did not show differential expression between young and old gingival tissues (Fig 3B). This indicates that IL1B should be considered a candidate regulator of MMPs in old gingival tissues.

### Distinctive MMP and IL1 gene expression patterns in aged gingival fibroblasts

Fibroblasts are the main cell type responsible for the synthesis of periodontal connective tissue [3]. GFs have been shown to undergo age-dependent modifications in their size, their mitotic activity [16], and the synthesis and maintenance of extracellular matrix constituents [17]. In addition, resident GFs are known to secret MMPs [18].

**Fig 4:**
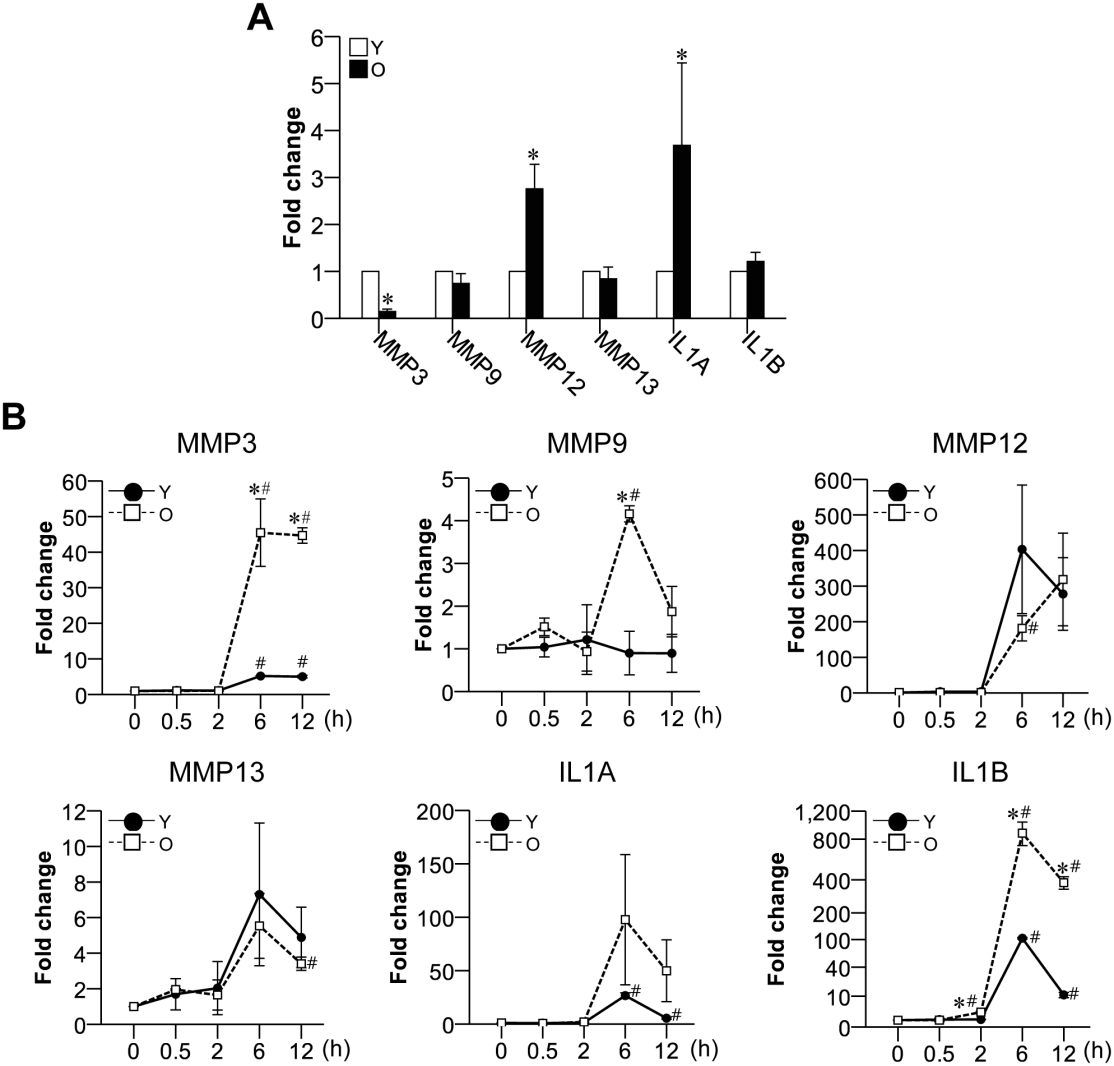
Expression levels of the mRNAs for MMP and IL1 family members in aged hGFs with and without oral bacterial infection. (A) The mRNA expression levels of MMPs and IL1 family members in young and old hGFs were evaluated; n = 3; *p < 0.05 versus the results from young hGFs. (B) Young and old hGFs were infected with *F*. *nucleatum* at a MOI = 10 for 0.5, 2, 6, 12 h, and the mRNA expression levels of MMP and IL1 family members were evaluated. Y, young hGFs; O, old hGFs; n = 3; *p < 0.05 versus Y within a same incubation time; and ^#^*p* < 0.05 versus the results obtained from the same cells at 0 h.

We thus investigated the mRNA expression patterns of MMPs and IL1 in primary cultured young and old hGFs to determine: i) whether aging induces molecular alterations among MMPs and IL1 in GFs; and ii) whether aged hGFs could be a main contributor for the upregulation of MMPs and IL1 observed in aged gingival tissues. Indeed, we found that the mRNA expression levels of MMP12 and IL1A were upregulated in old hGFs compared to young hGFs (Fig 4A). This suggests that the upregulations of MMP12 and IL1A in old hGFs could be critical factors responsible for natural gingival aging. In contrast, MMP3 was downregulated, and no change was seen in MMP9, MMP13, or IL1B in old hGFs compared to young hGFs (Fig 4A).

### Highly stimulated MMP and IL1 gene expression of aged hGFs to oral bacterial infection

The oral cavity is widely exposed to various pathogens and medications throughout life, and this can lead to periodontal attachment loss and bone destruction. The susceptibility to microbes and the severity of periodontal disease increase with age [1, 19]. In addition, oral bacteria and their products, such as lipopolysaccharide (LPS), can induced GFs to release inflammatory cytokines, including IL1B [20]. We hypothesized that the upregulations of the mRNAs encoding MMPs and IL1B in aged gingival tissues may reflect the increased susceptibility and weakened defense ability of an aged host against a variety of stimulants, such as bacteria.

To test this hypothesis, we evaluated target gene expression following the infection of young and old hGFs with the oral bacterium, *F*. *nucleatum*. Compared to young hGFs, old hGFs showed a highly enhanced upregulation to mRNA expressions of MMP3, MMP9 (both at 6 h) and IL1B (at 2 h) after infection with *F*. *nucleatum* (Fig 4B). The mRNAs for MMP3 and IL1B were also upregulated in young hGFs at 6 h post-infection, compared to 0 h, but these inductions were smaller and delayed compared to those observed in old hGFs (Fig 4B). We propose that the higher bacterium-induced mRNA inductions of the MMP3, MMP9, and IL1B in old hGFs may reflect age-related alterations in the response of these cells to microbial pathogens. Together, our novel results suggest that MMP3, MMP9, and/or IL1B may represent markers for externally-induced molecular changes in aged gingival tissues.

## Discussion

We herein addressed the genetics of gingival aging by using RNA sequencing to comprehensively and quantitatively compare differential gene expression between healthy gingiva of young and aged subjects. We also the first offer the assessment of aging-related changes in MMP expression in human gingival tissue, although several papers reported inflammation-related MMP expression in hGF [21, 22] and MMP9 regulation during *in vitro* aging in rat GF [23].

Our function annotation of the DEGs demonstrated that gingival aging was associated with cancer-related diseases and bio-functions followed by organismal injury and abnormalities, and reproductive system disease (Fig 1C). Some of the biologic mechanisms that regulate aging may be involved in the pathogenesis of diseases that increase with age, such as cancer [24, 25]. In the present study, some of the elderly donors had systematic diseases or disorders including cancer, osteoporosis, or hypertension (S1 Table). Thus, although their gingiva were healthy, it is formally possible that the gene expression profiles reported herein may reflect systematic conditions of the donors. Moreover, they could reflect a general aged phenotype, rather than one specific to the gingiva.

MMPs and their tissue inhibitors, the TIMPs, constitute an important proteolytic pathway that affects the remodeling of tissues and the structure/composition of the extracellular matrix (ECM) [26]. Age-dependent alterations in MMPs and TIMPs have been studied in skin [27] and cardiovascular disease [26], and increased activities of MMP2 and MMP9 have been associated with tenocyte aging [28]. Additionally, the mRNA levels of MMP2, MMP8 and TIMP1, but not TIMP2, were increased by aging in periodontal ligament cells (PDLCs) [10]. A recent study identified significant aging-related deficiencies in the wound healing processes of gingival tissues, including decreases in the cell migration and collagen gel remodeling of aged fibroblasts [29]. Similarly, we herein found aging-related upregulations of specific MMPs in aged gingiva, but no change in any TIMP.

MMP27 was the only MMP that our real-time PCR identified as being downregulated in aged gingival tissues (Fig 2B). This is consistent with previous reports that MMP27 decreases with age in skin [30, 31]. MMP27 is known to be expressed in endometrial macrophages related to menstruation and ovarian/peritoneal endometrial lesions [32] and thus is likely to show the expression accompanied by hormonal cycle [33]. We not know for certain why MMP27 is decreased in aged gingiva, but we speculate that age-related reductions in hormone levels might decrease the tissue expression of MMP27. Further studies are needed to determine whether MMP27 decreases in all tissues as age increases and/or hormone levels decrease.

Increasing age could be a potential risk factor for periodontal disease [3]. Some loss of periodontal attachment and alveolar bone may be expected in older persons, but in healthy adults, age alone does not lead to a critical loss of periodontal support [3, 34]. A previous study showed that cerebral MMP12 is upregulated during aging, and that this enhances aging-associated neuro-inflammation in the mouse brain [35]. Thus, MMP12 may contribute to inflammatory diseases during the aging process. MMP12 also appears to play a possible role in the natural aging of gingiva, as we found that it is upregulated during *in vitro* aging of control (non-infected) hGFs (Fig 4A). Meanwhile, the high-level stimulations of MMP3 and MMP9 in old hGFs subjected to bacterial infection may reflect a stress-induced aging phenotype in the gingiva (Fig 4B).

The immune system undergoes profound age-related changes, including gradual increases in the production and circulation of proinflammatory cytokines [36]. The expression level of IL1B is positively correlated with attachment loss [37], and this cytokine is associated with continuous tissue destruction in periodontitis [38]. Previous studies described that IL1 is: i) upregulated in gingival tissues from periodontitis patients [39]; ii) produced in aged human diploid fibroblasts [40]; iii) increased at the mRNA and protein levels by LPS-mediated stimulation of old hGFs [41]; and iv) capable of triggering MMP production in resident gingival fibroblasts [18, 42]. Similar to these results, we herein report that IL1B appears to be associated with age-related inflammatory processes in the gingiva. Biologic activity of IL1B requires proteolytic processing for activation by IL1B-converting enzyme (ICE or caspase-1), generally known as the primary physiological protease [43]. However, the presence of active IL1B in ICE-null mice indicated the possible mechanisms of IL1B activation by other proteases [44]. As a caspase-1-independent mechanisms of IL-1β activation, several studies showed that MMPs (MMP2, -3, and -9) activate the IL1B precursor to the active form [45]. Interestingly, MMPs (MMP1, -2, -3, and -9) can degrade the mature IL1B [46]. It suggested potentially dual roles for MMPs that MMPs can either promote or repress inflammation by the direct proteolytic processing of inflammatory cytokines and chemokines to activate, inactivate, or antagonize chemokine function [38]. Additionally, several studies have described that specific MMPs control chemokines activity [47, 48]. Thus, identifying specific MMP-cytokine and -chemokine regulations would provide an information to inhibit potentially detrimental and specific processes that are associated with gingival aging. Further work is needed to determine whether IL1B contributes to the prominent inductions of MMP3 and MMP9 in aged gingiva subjected to inflamm-aging [49] and/or is stimulated by excess secretion of specific MMPs associated with gingival aging.

In conclusion, we believe this is the first report of the RNA sequencing-based genetic profiling of young and old human gingival tissues. Our findings suggest that MMPs (MMP3, -9, -12, and -13) and IL1B may be relevant factors in gingival aging and/or stress-induced aging. Our findings provide a first step towards a quantitative and comprehensive understanding of the changes that occur in the transcriptome during gingival aging. In the future, this may enable the identification of candidate markers and/or therapeutic targets for gingival aging.

## Supporting Information

S1 Fig Sample correlation in a group. Scatter plots matrices and correlation coefficient (*r* value) in all samples.
(TIF)

S1 Table Patient characteristics and gingival status. (DOC)

S2 Table Gene list and primer sequences for quantitative real-time PCR. (DOC)

S3 Table Mapping rate to reference genome. (DOC)

S4 Table Full list of genes showing significantly differential expression between young and old gingival tissues. (XLSX)

S5 Table Full list of canonical pathways, disease and bio functions for DEGs between young and old gingival tissues. The lists are shown in each sheet as follows: S5.1 Table. Full list of canonical pathway for DEGs between young and old gingival tissues. S5.2 Table. Full list of disease and bio functions for DEGs between young and old gingival tissues.
(XLSX)
