# Supplementary figures and images for: Differential Matrix Metalloprotease (MMP) Expression Profiles Found in Aged Gingiva
Source: PLoS One. 2016 Jul 8;11(7):e0158777. doi: 10.1371/journal.pone.0158777 (PMC4938517; doi:10.1371/journal.pone.0158777)

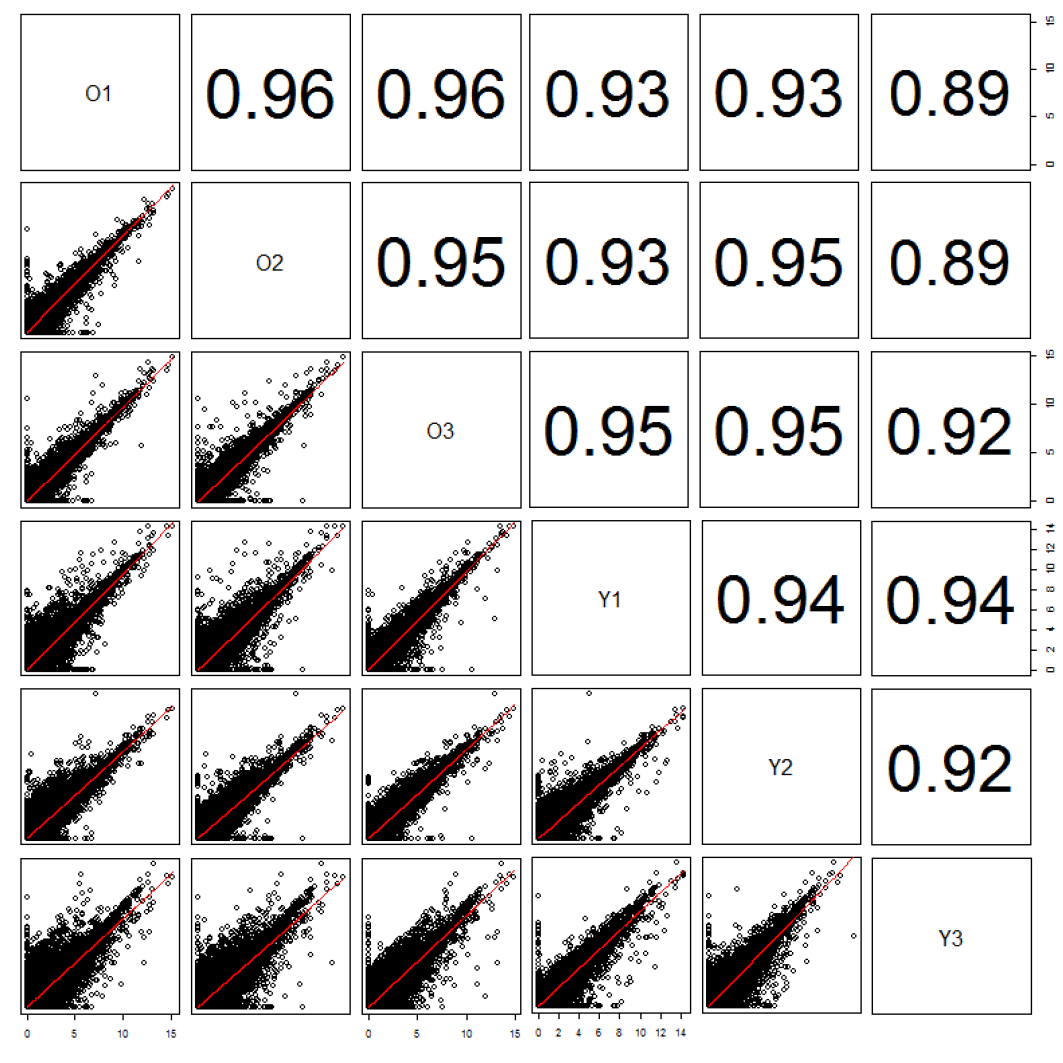

Supplement: S1 Fig — Scatter plots matrices and correlation coefficient (r value) in all samples. (TIF) [file pone.0158777.s001.tif]
