# Supplementary material for: Differential Matrix Metalloprotease (MMP) Expression Profiles Found in Aged Gingiva
Source: PLoS One. 2016 Jul 8;11(7):e0158777. doi: 10.1371/journal.pone.0158777 (PMC4938517; doi:10.1371/journal.pone.0158777)
Supplement: S1 Table — (DOC) [file pone.0158777.s002.doc]

**Table S1. Patient characteristics and gingival status.**

| Patient | Gender | Age (years) | Gingival status (0-3)^a^ | Systematic disease/or disorder | Sample  Group | Purpose of use |
| --- | --- | --- | --- | --- | --- | --- |
| 1 | F | 17 | 0 | None | Young | RNA-seq. |
| 2 | F | 19 | 0 | None | Young | RNA-seq. |
| 3 | F | 19 | 0 | None | Young | RNA-seq. |
| 4 | F | 20 | 0 | None | Young | Real-time PCR |
| 5 | F | 20 | 0 | None | Young | Real-time PCR |
| 6 | F | 21 | 0 | None | Young | Real-time PCR |
| 7 | F | 20 | 0 | None | Young | Real-time PCR |
| 8 | F | 20 | 0 | None | Young | Real-time PCR |
| 9 | M | 19 | 0 | None | Young | MMP activity |
| 10 | M | 20 | 0 | None | Young | MMP activity |
| 11 | M | 20 | 0 | None | Young | MMP activity |
| 12 | F | 64 | 0 | Liver cancer | Old | RNA-seq. |
| 13 | F | 66 | 0 | Osteoporosis | Old | RNA-seq. |
| 14 | F | 66 | 0 | Hypertension | Old | RNA-seq. |
| 15 | F | 71 | 0 | None | Old | Real-time PCR |
| 16 | F | 75 | 0 | Aplastic anemia | Old | Real-time PCR |
| 17 | F | 80 | 0 | Lung cancer | Old | Real-time PCR |
| 18 | F | 83 | 0 | None | Old | Real-time PCR |
| 19 | F | 86 | 1 | Colorectal cancer | Old | Real-time PCR |
| 20 | M | 62 | 0 | None | Old | MMP activity |
| 21 | M | 64 | 0 | Cancer | Old | MMP activity |
| 22 | M | 74 | 0 | None | Old | MMP activity |

^a^0 = no evidence of inflammation, 1 = mild inflammation, 2 = moderate inflammation and 3 = severe inflammation
