# Supplementary material for: Differential Matrix Metalloprotease (MMP) Expression Profiles Found in Aged Gingiva
Source: PLoS One. 2016 Jul 8;11(7):e0158777. doi: 10.1371/journal.pone.0158777 (PMC4938517; doi:10.1371/journal.pone.0158777)
Supplement: S2 Table — (DOC) [file pone.0158777.s003.doc]

**Table S2. Gene list and primer sequences for quantitative real-time PCR.**

| **Gene name** | **Direction** | **Sequence** |
| --- | --- | --- |
| MMP3 | Reverse  Forward | 5’- TGACCCAAATGCAAAGAAAGTG -3’  5’- TGCCTTCTACATATCTCTTTCAACAATT -3’ |
| MMP9 | Reverse  Forward | 5’- GGGCTCCCGTCCTGCTT -3’  5’- TCCTCCCTTTCCTCCAGAACA -3’ |
| MMP10 | Reverse  Forward | 5’- ACCTCCCCCTGCCTCTACTG -3’  5’- CACACTTGGCTGGCATCTCA -3’ |
| MMP12 | Reverse  Forward | 5’- TGGTTTGGTTGTTAGAAATGGTGTA -3’  5’- CTGAGGACATAGCAAATATGCAATAAA -3’ |
| MMP13 | Reverse  Forward | 5’- GGAGAAAGCTTGGTTCTGTGAAC -3’  5’- TCCAGCCACGCATAGTCATATAGAT -3’ |
| MMP11 | Reverse  Forward | 5’- GGGTGCCCTCTGAGATCGA -3’  5’- CTTCACAGGGTCAAACTTCCAGTA -3’ |
| MMP16 | Reverse  Forward | 5’- CTATTCTTCGTCGTGAGATGTTTGTT -3’  5’- AGTAAGTAATTTGCATTGGGTATCCA -3’ |
| MMP27 | Reverse  Forward | 5’- CTGGGTGGTGACACTCATTTTG -3’  5’- AGCAGCCACAAGAAACAAGTTG -3’ |
| TIMP2 | Reverse  Forward | 5’- AACGCGTGGCCTATGCA -3’  5’- ACGGGAGACGAATGAAAGCA -3’ |
| TIMP3 | Reverse  Forward | 5’- GCTTCCCTTGGACACTAACTCTTC -3’  5’- CCCCATATGACAGCATAGACCTT -3’ |
| RECK | Reverse  Forward | 5’- GAATGCTCCTCCACCTCACTCT -3’  5’- CCTTTCCTTGGCCAATATTCAA -3’ |
| SDC2 | Reverse  Forward | 5’- GGAGTTATTGGCTTTCTCTTTGCA -3’  5’- TCATAGCTTCCTTCATCCTTCTTTC -3’ |
| THBS2 | Reverse  Forward | 5’- ATCGCTGTAGGTTTTGACGAGTTT -3’  5’- TAGTCGTCGTCCCGGTCAGT -3’ |
| ADAM12 | Reverse  Forward | 5’- TCAATTCTGCAGGTGATCCTTATG -3’  5’- TTAGCATCTCTCATCTCGCATTTG -3’ |
| IL1A | Reverse  Forward | 5’- CGCCAATGACTCAGAGGAAGA -3’  5’- TCCTCATAAAGTTGTATTTCACATTGC -3’ |
| **Gene name** | **Direction** | **Sequence** |
| IL1B | Reverse  Forward | 5’- CGGCCAGGATATAACTGACTTCA -3’  5’- CCACATTCAGCACAGGACTCTCT -3’ |
| JNK1 | Reverse  Forward | 5’- GAGGAGAGAACCAAGAATGGAGTTA -3’  5’- CGACGATGATGATGGATGCT -3’ |
| JNK2 | Reverse  Forward | 5’- CTCTGCGTCACCCATACATCA -3’  5’- TTCCAACTGGGCATCATAAATTT -3’ |
| PDGFB | Reverse  Forward | 5’- AAGCACCGGAAATTCAAGCA -3’  5’- ATATTAAATAACCCTGCCCACACACT -3’ |
| β-Actin | Reverse  Forward | 5’- AACTTGCGCAGAAAACAAGATG -3’  5’- TTTTTAAATCCTGAGTCAAGCCAAA -3’ |
