# Supplementary material for: Differential Matrix Metalloprotease (MMP) Expression Profiles Found in Aged Gingiva
Source: PLoS One. 2016 Jul 8;11(7):e0158777. doi: 10.1371/journal.pone.0158777 (PMC4938517; doi:10.1371/journal.pone.0158777)
Supplement: S3 Table — (DOC) [file pone.0158777.s004.doc]

**Table S3. Mapping rate to reference genome.**

| Sample group^a^ | Left reads (counts) | Right reads  (counts) | Overall read mapping rate (%)^b^ | Aligned pair reads (counts) | Concordant pair alignment rate (%)^c^ |
| --- | --- | --- | --- | --- | --- |
| Y1 | 8,285,098 | 8,285,098 | 89.0% | 6,868,365 | 82.3% |
| Y2 | 11,751,141 | 11,751,141 | 89.4% | 9,829,448 | 82.9% |
| Y3 | 10,852,245 | 10,852,245 | 88.9% | 9,009,654 | 82.3% |
| O1 | 13,346,843 | 13,346,843 | 89.1% | 11,187,729 | 83.2% |
| O2 | 11,416,231 | 11,416,231 | 89.1% | 9,536,028 | 82.9% |
| O3 | 10,333,753 | 10,333,753 | 88.6% | 8,534,814 | 82.1% |

^a^Y and O indicate young and old gingival tissues, respectively. Three biological replicates were used.

^b^The rate matched with left or right reads of paired-end sequencing

^c^The rate matched with two of both (left and right) reads of paired-end sequencing
